# The (unresolved) antibody paradox

**DOI:** 10.1172/JCI183886

**Published:** 2024-07-15

**Authors:** Subashan Vadibeler

**Affiliations:** Leeds Institute of Medical Research, University of Leeds, Leeds, United Kingdom; Department of Oncology, University of Oxford, Oxford, United Kingdom.

I was a keen first-year medical student in Kuala Lumpur, standing in a pediatric bay. “Isles of white in a sea of red,” my consultant said while inspecting the child’s forearms, flushed bright red with faint white spots. Despite the angry rash, the child was in recovery after being admitted with a high fever, aches, and vomiting. Here, I first saw the immune-related dengue rash; a clue that my patient had escaped severe dengue that affects about 10% of those infected (1). Like the rash, the disease which causes it, dengue fever, is a tropical viral illness full of paradoxes.

I soon stumbled upon another immunological conundrum in dengue that goes deeper than the skin. I discovered that the child’s risk of severe dengue would be higher if they were reinfected in the future. This risk would be further heightened if the reinfection were caused by a different version of the same virus, of which four distinct types exist. Once again, I was faced with a contradiction. This increased risk of severe dengue during a secondary infection is counterintuitive to a central tenet of our immune system: memory. In theory, previous infections should train our immune system to respond better and faster and to have a stronger response to a familiar virus, but that was not always the case for dengue. Additionally, some infants, whose mothers had contracted dengue fever during pregnancy, also had a greater risk of developing severe dengue. These observations implicated antibodies as a possible cause. Virus-specific antibodies obtained either from a previous infection or from the mother through placental transfer could worsen dengue through a phenomenon known as antibody-dependent enhancement (2).

At present, not much is known about why antibody-dependent enhancement only occurs in some patients and under specific circumstances depending on the type, concentration, and quality of circulating antibodies. In fact, the world’s first dengue vaccine acted as a jarring reminder that vaccine-induced antibodies are also capable of disease enhancement. During its mass rollout in the Philippines in 2016, the risk of hospitalization and death from a subsequent infection after vaccination was higher in children under nine years of age (3), who likely have never had a natural infection. There has been an understanding regarding antibody-dependent enhancement in dengue since 1970 (4); given this, how was this adverse outcome not better predicted? As a result, not only were some children deprived of a vaccine that could prevent dengue-related deaths in the region, but general efforts to improve vaccine acceptance and eradicate vaccine-preventable diseases were also compromised. Sales and distribution of the dengue vaccine were halted, but the damage was already done. Vaccine confidence in Southeast Asia plummeted (5).

The challenges of antibody-dependent enhancement resurfaced during the COVID-19 pandemic, as predicting this phenomenon in new diseases can be difficult with our incomplete understanding. Amid debate about its occurrence in COVID-19, scientists at the Jenner Institute ran additional safety assessments while developing the Oxford-AstraZeneca COVID-19 vaccine, including preclinical testing in primates to ensure that disease enhancement did not occur with COVID-19 vaccine-induced antibodies (6). Besides these costly and time-consuming vaccine safety measures, uncertainties about antibody-dependent enhancement in COVID-19 also restricted the clinical use of experimental antibody-based strategies such as intravenous immunoglobulins (7). Despite these rigorous precautionary measures, antibody-dependent enhancement still became a tool heavily weaponized by antivaccination conspirators to fuel misinformation and vaccine hesitancy during the pandemic.

Besides the scientific and moral imperatives of better understanding antibody-dependent enhancement for emerging infectious diseases, there are also financial and economic incentives for studying this phenomenon. Antibody-based therapies have revolutionized not only the treatment of cancer and inflammatory, infectious, and autoimmune diseases, but also common conditions like asthma and osteoporosis, with over 150 antibody-based agents approved for use around the world (8). In 2023, the pharmaceutical sales of pembrolizumab, a therapeutic anticancer antibody, had once again surpassed COVID-19 vaccines in global revenue, becoming the highest grossing product on the market (9). Hefty investments are being pumped into antibody engineering efforts to harness all aspects of this incredible molecule to maximize its therapeutic potential. Therefore, poorly characterized antibody features like antibody-dependent enhancement can provide valuable insights into antibody biology that may open doors to more innovative clinical translations.

Antibody-dependent enhancement is no longer a field-specific question in dengue, but one that affects wider immunology, bioengineering, vaccinology, pharmacotherapeutics, public health, and pandemic preparedness. Our scientific rigor and funding need to appropriately reflect this broader relevance, especially in the context of climate change where tropical infectious diseases may soon affect a larger population (10). Importantly, we urgently need commercial assays to rapidly detect immune enhancement in novel pathogens and models to better predict clinical severity in patients who develop this phenomenon. Soon, we may even be able to unlock the overexuberant immune activation potential of antibody-dependent enhancement to our own therapeutic advantage in cancer vaccines. At its crux, these applications circle back to the fundamental question that remains unanswered: what is the exact cellular and molecular mechanism of antibody-dependent enhancement?

## Version 1 07/15/2024

Electronic publication
